# Reductive Modification of Carbon Nitride Structure by Metals—The Influence on Structure and Photocatalytic Hydrogen Evolution

**DOI:** 10.3390/ma15030710

**Published:** 2022-01-18

**Authors:** Emilia Alwin, Robert Wojcieszak, Kamila Kočí, Miroslava Edelmannová, Michał Zieliński, Agata Suchora, Tomasz Pędziński, Mariusz Pietrowski

**Affiliations:** 1 Faculty of Chemistry, Adam Mickiewicz University, Poznań, Uniwersytetu Poznańskiego 8, 61-614 Poznan, Poland; emilia.alwin@amu.edu.pl (E.A.); mardok@amu.edu.pl (M.Z.); agasuc@amu.edu.pl (A.S.); tomekp@amu.edu.pl (T.P.); 2 Univ. Lille, CNRS, Centrale Lille, Univ. Artois, UMR 8181-UCCS-Unité de Catalyse et Chimie du Solide, F-59000 Lille, France; robert.wojcieszak@univ-lille.fr; 3 Institute of Environmental Technology, CEET, VSB-Technical University of Ostrava, 17. listopadu 15/2172, 70800 Ostrava-Poruba, Czech Republic; kamila.koci@vsb.cz (K.K.); miroslava.edelmannova@vsb.cz (M.E.); 4 Centre for Advanced Technologies, Adam Mickiewicz University, Poznań, Uniwersytetu Poznańskiego 10, 61-614 Poznan, Poland

**Keywords:** metal photocatalysts, graphitic carbon nitride, wet impregnation method, photocatalytic activity, physicochemical characterization

## Abstract

Pt, Ru, and Ir were introduced onto the surface of graphitic carbon nitride (g-C_3_N_4_) using the wet impregnation method. A reduction of these photocatalysts with hydrogen causes several changes, such as a significant increase in the specific surface area, a C/N atomic ratio, a number of defects in the crystalline structure of g-C_3_N_4_, and the contribution of nitrogen bound to the amino and imino groups. According to the X-ray photoelectron spectroscopy results, a transition layer is formed at the g-C_3_N_4_/metal nanoparticle interphase, which contains metal at a positive degree of oxidation bonded to nitrogen. These structural changes significantly enhanced the photocatalytic activity in the production of hydrogen through the water-splitting reaction. The activity of the platinum photocatalyst was 24 times greater than that of pristine g-C_3_N_4_. Moreover, the enhanced activity was attributed to significantly better separation of photogenerated electron–hole pairs on metal nanoparticles and structural distortions of g-C_3_N_4_.

## 1. Introduction

The growing demand for energy, gradual depletion of fossil fuels, and increasing efforts aimed at protecting the natural environment have stimulated the search for new green energy sources. One of them can be hydrogen obtained from photocatalytic reactions, such as the photodecomposition of methanol/water mixtures. Methanol acts as a sacrificial reagent to improve the hydrogen generation yield [1,2]. The direct mechanism of this process assumes the oxidation of methanol and water by electron holes. The reaction of methanol with electron holes leads to hydrogen ions (H^+^) and hydroxyalkyl radicals (**^∙^**CH_2_OH) that undergo further reaction to give hydrogen cations and electrons. The reaction of water with electron holes leads to the generation of oxygen and protons. At the final stage, the protons are reduced by electrons, which leads to hydrogen release. Details on the mechanism of this reaction have been described by many authors [1,3,4,5,6]. Generally, one of the best-known photocatalysts, titania (TiO_2_), is used in this reaction [4,7,8,9]. However, this photocatalyst has limitations, such as a large band gap (anatase: 3.2 eV, rutile: 3.0 eV) [10]; hence, it is activated by ultraviolet (UV) radiation. Another drawback of TiO_2_ is the fast recombination of generated electrons and holes. Considering these limitations, new photocatalysts for solar-light water splitting that are free from such disadvantages should be developed.

Graphitic carbon nitride (g-C_3_N_4_) is a promising alternative to TiO_2_ [11,12]. First, it has a significantly smaller band gap (2.7 eV) than TiO_2_; thus, it can be activated by visible light. Moreover, it shows reliable thermal and chemical resistance, nontoxicity, and water resistance. In addition, the production by condensation of simple nitrogen-rich compounds such as urea [13,14], thiourea [15], melamine [16,17], and dicyandiamide (DCDA) [18] is easy and cheap. Its additional advantage is that, depending on the type of precursor and condensation conditions, final carbon nitride can have different physicochemical properties [19]. Since the first reports on the photocatalytic properties of g-C_3_N_4_ [20], attempts have been made to enhance its effectiveness, mainly by the addition of heteroatoms [21,22]. However, it has been discovered that better effects can be achieved by the introduction of noble metals on the carbon nitride surface. Li et al. reported a significant increase in the g-C_3_N_4_ activity during water splitting under visible light after the introduction of 2 wt.% Pt [23]. The positive effect of Pt on g-C_3_N_4_ was explained by the accumulation of electrons on the metal particle surface, which hindered the recombination of photogenerated electron–hole pairs [23]. Similar conclusions were drawn by Khan et al. [24] who claimed that the Pt particles on g-C_3_N_4_ act as electron trap centers.

Following these ideas, in this study, the effects of introducing Pt-group metals (Pt, Ru, and Ir) on the g-C_3_N_4_ surface were investigated. However, instead of the commonly used photoreduction or chemical reduction, the photocatalysts were synthesized using the wet impregnation method with an excess of solvent. The photocatalysts were then reduced with hydrogen to generate structural defects as a result of partial hydrogenation/hydrogenolysis of C−N bonds, which was expected to improve the separation of photogenerated electron–hole pairs and, consequently, the photocatalytic activity.

## 2. Materials and Methods

Graphitic carbon nitride was prepared by the pyrolysis of dicyandiamide (DCDA) (Sigma-Aldrich, Darmstadt, Germany, 99%) in a semi-closed system. In a 50-mL quartz crucible with a cover, 4 g of DCDA precursor was added and heated at 600 °C in a furnace for 4 h with a heating rate of 10 °C min^−1^ under ambient pressure in the air. After cooling to room temperature in the furnace, the obtained yellow material was ground into a fine powder in an agate mortar and labeled as CN.

Metallic (Pt, Ru, and Ir) photocatalysts were synthesized using the wet impregnation method. In 25 mL of water solutions of H_2_PtCl_6_, H_2_IrCl_6_, and methanolic solution of Ru_3_(CO)_12_, 2 g of the graphitic carbon nitride powder (CN) was dispersed. The metal loading in the photocatalysts was 0.5 and 1.0 wt.%. The suspension was stirred for 2 h at room temperature (only for Ru_3_(CO)_12_ at 40 °C) and was subsequently evaporated. The samples were dried overnight at 80 °C and reduced at 300 °C for 4 h at a heating rate of 10 °C min^−1^ under ambient pressure in the presence of pure hydrogen. Because the reduction can influence the g-C_3_N_4_ structure, it was reduced under the same conditions as those of the photocatalysts and was designated as CNr.

The photocatalysts were characterized by low-temperature N_2_ adsorption, X-ray diffraction analysis (XRD), elemental analysis (EA), transmission electron microscopy (TEM), scanning electron microscopy (SEM), ultraviolet-visible diffuse reflectance spectroscopy (UV-Vis), photoluminescence spectroscopy (PL), and X-ray photoelectron spectroscopy (XPS).

The specific surface area (SSA) was determined by the Brunauer–Emmett–Teller (BET) method using a Micromeritics ASAP 2010 (Micromeritics, Norcross, GA, USA) surface area and porosity analyzer (surface areas were obtained from N_2_-adsorption isotherms collected at 77 K). Cumulative pore volume and pore diameter were determined by Barrett–Joyner–Halenda (BJH) method from the desorption branch of isoterm.

The metal loading in the photocatalysts after reduction was determined using the inductively coupled plasma (ICP) method. An Agilent 720-ES ICP (Santa Clara, CA, USA) optical emission spectrometer combined with a Vulcan 42S robot was used to determine the metal loading. The measured metal contents were close to those assumed and amounted to 0.86, 0.82, and 0.90 wt.% for Pt, Ru, and Ir, respectively.

A Hitachi HT7700 microscope (Hitachi, Tokyo, Japan) at an accelerating voltage of 100 kV was used to record TEM images. The particle size distribution histograms were calculated from 394 particles for Pt, 338 for Ru, and 172 for Ir using the ImageJ program (v.1.53e, 2020) developed at National Institutes of Health and the Laboratory for Optical and Computational Instrumentation (LOCI, University of Wisconsin, Madison, WI, USA). An FEI Helios NanoLab 660 (Thermo Fisher Scientific, Waltham, MA, USA) electron microscope was used to obtain SEM images.

The XRD analysis was performed in the 2θ range between 6° and 40° on a Bruker D8 Advance diffractometer (Billerica, MA, USA) using CuKα radiation. Bragg’s law nλ = 2dsinθ (where n is an integer, λ is the radiation wavelength (λ = 1.5418 Å), and θ is the reflection angle for the reflex hkl) was used to calculate the distance d_hkl_ for the sample. The crystallite size of g-C_3_N_4_ was calculated using the Scherrer formula D = Kλ/βcosθ, where D is the crystallite size in nm, K is the Scherrer constant (0.94), λ is the radiation wavelength (λ = 1.5418 Å), and β is the full width of the (002) crystallite peak at half maximum.

The elemental analysis was performed using a Flash 2000 exhaust gas analyzer (Thermo Fisher Scientific, Waltham, MA, USA) by combustion at 900–1000 °C.

The UV-Vis diffuse reflectance spectra were recorded on a Jasco (Tokyo, Japan) model V-670 spectrophotometer. A Jasco (Tokyo, Japan) spectrofluorometer model FP-8300 using an excitation source of 350 nm was employed for PL spectroscopy.

The XPS analysis of the carbon nitrides was performed using a Kratos Axis Ultra spectrometer (Kratos Analytical, Manchester, UK). The excitation source was a monochromatized aluminum X-ray source (Al Kα (1486.6 eV) operated at 10 mA and 15 kV. The charge referencing method used was the C (C, H) component of the C 1s peak of adventitious carbon fixed at 284.5 eV. Spectroscopic data were processed by CasaXPS ver. 2.3.17PR1.1 software (Casa Software Ltd., Teignmouth, UK) using a peak-fitting routine with Shirley background and asymmetrical Voigt functions.

Photocatalytic tests were performed in a stainless-steel batch-mixed photoreactor (volume = 348 mL). Initially, helium saturated the reaction mixture, containing 100 mL of 50% methanol with a photocatalyst (0.1 g), to purge the air. A UV LED lamp (365 nm wavelength; M365LP1, Thorlabs, Bergkirchen, Germany) was the source of irradiation and was placed on a quartz glass window on the top of the photoreactor in a horizontal position. Before the start of the reaction, a gaseous sample was taken (at time 0 h) through the septum using a syringe. All gaseous samples were analyzed using a gas chromatograph (Shimadzu Tracera GC-2010Plus, Kyoto, Japan) equipped with a barrier discharge ionization detector. The reaction was performed for 4 h, and gaseous samples were extracted at 1, 2, 3, and 4 h for analysis. Each test was conducted twice with the same photocatalyst to determine at least short-time stability and reusability.

## 3. Results and Discussion

### 3.1. Photocatalytic Activity

One of the factors limiting the photocatalytic activity of semiconductors is the fast charge recombination. To restrict recombination, an electron-donating substance, such as a sacrificial agent, is added. This substance supplies the system with electrons and simultaneously binds the electron holes, which results in better charge separation and enhances the efficiency of the water reduction process. In general, such sacrificial agents are triethanolamine, methanol, ethanol, or aliphatic or aromatic compounds. Methanol is one of the most commonly used solvents for industrial applications; however, it can have harmful effects on the environment. Therefore, as a better option, it can be utilized as a sacrificial agent in the photocatalytic decomposition of water [25]. Our earlier results [19] suggested that g-C_3_N_4_ can be an interesting alternative to the commonly used photocatalysts in water/methanol decomposition.

**Figure 1:**
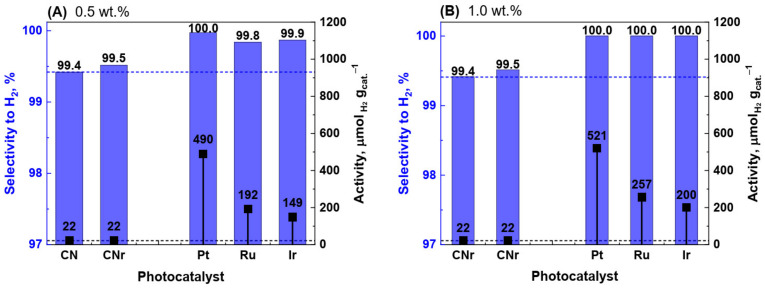
Photocatalytic activity of carbon nitride (CN), reduced carbon nitride (CNr), and metallic photocatalysts in the decomposition of water/methanol solution after 4 h of irradiation at different metal loadings: (**A**) 0.5 wt.% and (**B**) 1.0 wt.%. Black points and blue bars represent photocatalytic activity and selectivity to hydrogen, respectively.

Figure 1A shows the activity and selectivity of CN, CNr, and metallic photocatalysts. The activities and selectivities of CN and CNr were very similar. This indicates that the reduction of carbon nitride does not significantly affect its photocatalytic properties. The activity of CN and CNr in the photocatalytic decomposition of the water/methanol mixture was relatively low (22 µmol_H2_·g_cat_.^−1^) (Figure 1A). Therefore, 0.5 wt.% and 1.0 wt.% of each of the metals Pt, Ru, and Ir were introduced on the surface of CN using the conventional impregnation method, instead of the commonly used photoreduction or chemical reduction methods. Metals increase the separation between the photoinduced electrons and holes; thus, an increase in the activity was expected. The activities of all the photocatalysts increased significantly compared to those of CN and CNr, and the highest increase in the activity (for 0.5 wt.% catalysts), that is by approximately 22 times, was observed for Pt (Figure 1A). Moreover, the selectivity to hydrogen considerably increased from 99.4% for CN to 99.8–100.0% for metal-modified photocatalysts.

In the next logical step, the possibility of photocatalytic activity enhancement using a higher metal loading of 1.0 wt.% was investigated. The results are shown in Figure 1B. The most significant change was the increase in the selectivity to hydrogen. It reached 100% for all the metallic photocatalysts, while the selectivity for hydrogen using carbon nitride was 99.4%. The selectivity of 100% is undoubtedly a great advantage when considering applications of these photocatalysts in fuel cells. It is obvious that even the presence of trace amounts of CO in hydrogen fuel cells can be dangerous [26]. Along with the increase in hydrogen selectivity, the change in metal loading from 0.5 to 1.0 wt.% also increased the photocatalytic activity, as seen in Figure 1B. It should be mentioned that each photocatalytic test was conducted twice on the same photocatalyst sample and no decrease in activity or change in selectivity was observed. This proves the short-time stability and reusability of the photocatalysts. The significant increase in the photocatalytic activity of the Pt photocatalyst (24 times higher than that of CNr) could be related to the physical and chemical changes in the structure, morphology, and composition of the photocatalyst. Generally, the standard physicochemical characterization is used in such cases to reveal the smallest changes in the photocatalysts and understand the factors responsible for the increase in their photocatalytic activity.

### 3.2. Surface Area and Porosity of Metal/CN Photocatalysts

**Figure 2:**
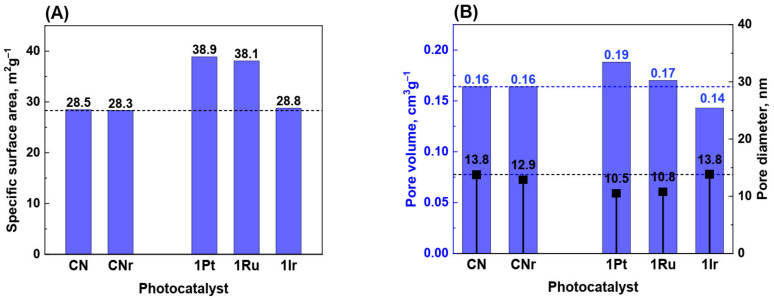
Specific surface area of carbon nitride and photocatalysts with 1.0 wt.% metal loading—(**A**). Cumulative pore volume and average pore size for 1.0 wt.% photocatalysts—(**B**).

In the impregnation method, the active phase covers the support surface. Consequently, the surface area after deposition of the active phase is expected to decrease compared to that of the pure support, because the active phase may block or fill the pores in the support. After the deposition of the Pt-group metals, the surface area of the photocatalyst increased, and the increase in the surface area was significant—36% for Pt/CN photocatalyst. Simultaneously, the reduction did not influence the SSA of g-C_3_N_4_. Furthermore, the surface area of the photocatalysts with higher metal loading (1.0 wt.%) was slightly higher than that of the photocatalysts with the metal loading of 0.5 wt.% (Figure 2A and Figure S1). This is inconsistent with the mechanism of pore blocking by the active phase.

What is the reason for such an increase in the surface area of the Pt-group metal photocatalysts? For active phase contents as low as 0.5 or 1.0 wt.%, the increase in the surface area cannot be assigned to the appearance of this phase (metal) in the catalytic system, thus it must be related to the structural changes in the support. An explanation could be proposed assuming that changes in the support structure occurred during the photocatalyst’s reduction was carried out at 300 °C for 4 h in the flow of hydrogen. In this process, the graphitic carbon nitride (organic polymer) is exposed to atomic hydrogen, an aggressive reducing agent, formed on the metal surface during reduction. Platinum-group metals are known for their high activity in hydrogenolysis and hydrogenation reactions. In the vicinity of metal particles, hydrogenolysis and hydrogenation of the C−N bonds can occur, leading to the partial etching of g-C_3_N_4_ [27]. Consequently, new pores and new structural defects are generated; hence, the SSA will increase. Indeed, the total surface area of the photocatalysts containing metals increased compared to that of the CNr support, as shown in Figure 2A and Figure S1. This increase is particularly noticeable for the photocatalysts with 1.0 wt.% of Pt and Ru.

Figure 2B shows the cumulative pore volume and average pore size for 1.0 wt.% photocatalysts. The decrease in the pore diameter is due to the increased contribution of small pores.

The porosity and SSA results clearly indicate that g-C_3_N_4_ must undergo structural changes during the photocatalytic reduction process, which leads to an increase in the SSA. However, these important modifications in the structure should also affect the results obtained using other characterization methods. This was confirmed by several techniques, as discussed below.

### 3.3. Electron Microscopy Analysis

**Figure 3:**
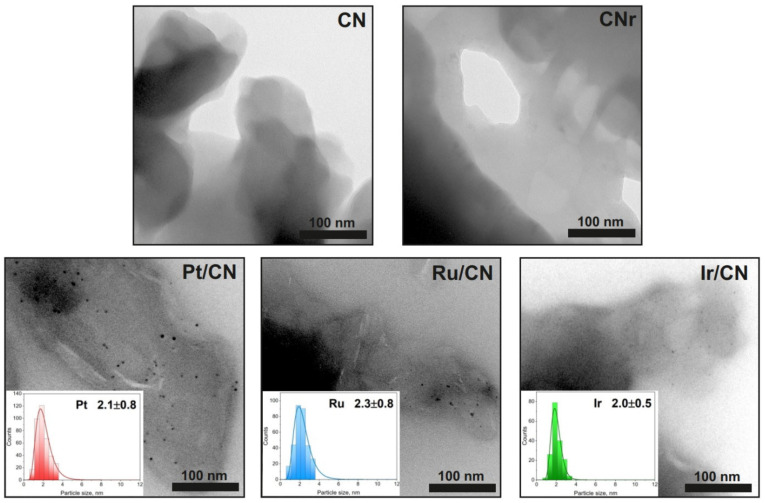
TEM images of CN, CNr, and metal photocatalysts (1.0 wt.%). The inset figures in the TEM images of the metal photocatalysts show the corresponding particle size distribution.

Figure 3 shows the TEM images of carbon nitride (before and after reduction) and the photocatalysts. The average size of the metal crystallites was determined from these images, and histograms for particle size distribution were prepared. The particle size distribution is narrow, and the size of the metallic particles is approximately 2 nm.

The SEM images did not show any noticeable differences in the structure of the supports (CN and CNr) and metallic photocatalysts (Figure S2). The images of all the photocatalysts show larger crystalline particles of g-C_3_N_4_, which have a layered structure and are frayed at the edges.

### 3.4. Powder Diffractometry of Metal/CN Catalysts

**Figure 4:**
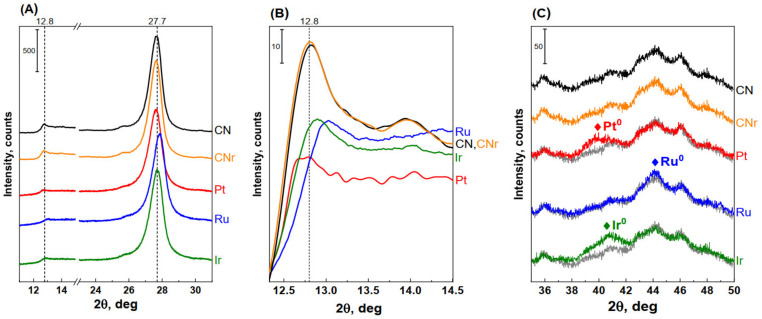
XRD patterns of pristine CN, CNr, and metallic photocatalysts (1.0 wt.%) at different 2θ angles: (**A**) 11°–31°, (**B**) 11.5°–14.5°, and (**C**) 35°–50°. The grey lines in (**C**) represent the XRD pattern of CNr, which was used as a reference for photocatalysts to highlight reflections from metals.

Powder diffractograms of CN, CNr, and the metal photocatalysts are shown in Figure 4A. Each diffractogram shows two characteristic reflections of carbon nitride. The first reflection (2θ = 27.7°) corresponds to the (002) crystallographic plane and indicates the interlayer stacking of aromatic rings. The second reflection (2θ = 12.8°) corresponds to the (210) plane and is related to the in-plane structural packing motif of tri-s-triazine units in melon (separation between parallel melon chains) [28,29,30,31]. The intensity of the reflection at ~27.7° (002) in the diffractograms of the photocatalysts is slightly lower than that of the support, and at the same time, its full width at half maximum (FWHM) increases. From the latter parameter, the size of the g-C_3_N_4_ crystallites was estimated using the Scherrer formula. For the metallic photocatalysts, the size of the g-C_3_N_4_ crystallites was slightly smaller than that of the CN (or CNr) support, as shown in Table 1. The number of layers composing the carbon nitride crystallite was calculated by dividing the size of g-C_3_N_4_ crystallites by the interplanar distance d. The number of layers was ~30 for CNr and ~29 for the metal photocatalysts (rounded up to unity). This indicates that the large-scale structure of carbon nitride undergoes slight destruction.

Figure 4B shows the diffractograms of the support and photocatalysts in the 2θ angle range of 11.5°–14.5°. The reflection corresponding to the plane (210) appears in this range, which is related to the in-plane structural packing motif of heptazine units. After the impregnation of carbon nitride with the metals, a significant decrease in the intensity of this reflection and its shift towards higher 2θ are observed. It indicates a slight decrease in the distance between the parallel melon chains. These changes are clearly notable. Figure 4C shows the diffractograms of the photocatalysts at the 2θ angle range (35°–50°) in which the reflections from the metallic phases appear. Such reflections were observed in diffractograms of all photocatalysts—they are wide, which implies a high dispersion of the metal phase.

In summary, the XRD results revealed changes in the structure of g-C_3_N_4_ as a result of the introduction of metals onto the g-C_3_N_4_ surface. In general, the changes can be described as an increase in the number of defects in the g-C_3_N_4_ support structure (a decrease in the degree of ordering).

### 3.5. Elemental Analysis

Pristine CN, CNr, and the metal photocatalysts were subjected to elemental analysis after their reduction to determine the contents of N, C, and H (Table 2). No noticeable differences in the elemental composition of CN and CNr were observed; therefore, Table 2 presents only the results for CNr. In general, the contents of C, N, and H in the photocatalysts were similar to those in CNr. However, one noticeable difference is that the carbon content remains unchanged with simultaneous reduction of nitrogen content. The decrease in the nitrogen content can be clearly observed from the C/N atomic ratio (Table 2). Depending on the catalyst, the C/N atomic ratio is higher than that of pristine CN by 4–8%. Apart from the above difference, the hydrogen content in all photocatalysts, except Pt, was higher than that in CNr.

### 3.6. UV-Vis and PL Spectral Analysis

**Figure 5:**
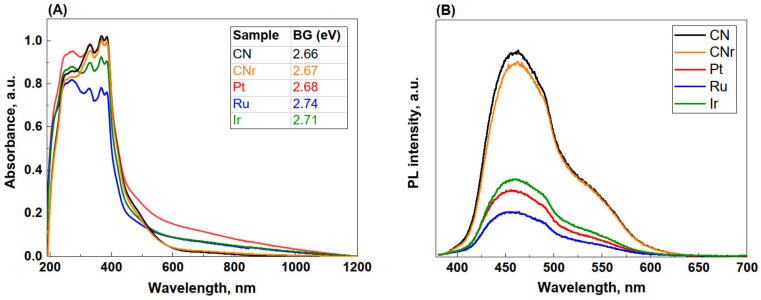
UV–Vis diffuse reflectance—(**A**) and PL spectra—(**B**) for CN, CNr, and metal photocatalysts (1.0 wt.%). Inset in (**A**) shows the band gap energies of the samples.

The UV-Vis spectra of the photocatalysts (Figure 5A) display a characteristic band in the range of 190–450 nm that corresponds to the aromatic structures in g-C_3_N_4_. All UV-Vis spectra show two characteristic peaks: the first at ~250 nm attributed to the π → π* transitions in aromatic rings, and the second at ~380 nm attributed to the electron transitions from the nonbonding orbitals of nitrogen atoms to the antibonding aromatic orbitals. An increase in absorption in the visible range above 600 nm was observed (known as the Urbach tails) for all photocatalysts [32,33]. Compared to CNr, metallic photocatalysts exhibit an apparently broader and stronger absorption tail extending to 1000 nm, which is attributed to the defect-related states located within the band gap [34]. The increased light absorption may generate a higher number of electron–hole pairs under visible light, resulting in further effective absorption of radiation by the photocatalysts and their increased activity.

As shown in Figure 5A, carbon nitrides have the highest absorption, particularly at 365 nm, at which the photocatalytic reactions were performed. This suggests that there must be another factor determining the photocatalyst activity besides the ability to absorb radiation. It should be considered that the added metals generate additional states within the band gap, diminishing the electron–hole recombination rate, which enhances the photocatalytic activity. The band gap size was determined from the Kubelka–Munk plot, illustrating the dependence of F(R)^2^ on the photon energy. The band gaps of the photocatalysts were determined as 2.68–2.74 eV and were all slightly greater than those obtained for CN and CNr (2.66 and 2.67 eV) (inset in Figure 5A). The increase in the band gap energy was earlier observed for ultra-low loading of Ru clusters over g-C_3_N_4_, and it was interpreted as related to the decreased degree of ordering of the g-C_3_N_4_ structure [35]. Furthermore, Maschmeyer et al. [36] demonstrated a relationship between the band gap increase and structural changes in g-C_3_N_4_ resulting from the high temperature (550 °C) treatment in hydrogen.

The PL emission in semiconductors, such as g-C_3_N_4_, results from the recombination of photoinduced electron–hole pairs. Hence, based on the PL intensity, we can conclude that the lower the PL intensity, the smaller is the recombination rate of the photogenerated electrons and holes. The differences in band intensity between CN and CNr were negligible, thereby indicating that the reduction did not alter the photoluminescent properties of carbon nitride. A strong (greater than three folds) PL emission quenching for all metal-loaded samples compared to that of CNr was observed (Figure 5B). Metallic sites act as temporary electron-trapping sites where electrons from the conduction band of g-C_3_N_4_ are trapped before recombination with the holes in the valence band [37]. As a result, significantly fast recombination of electrons with holes is prevented and the lifetime of the photoinduced electron–hole pairs is extended, which contributes to an increase in the photocatalytic activity.

### 3.7. XPS Analysis

X-ray photoelectron spectroscopy is an extremely useful technique for the advanced characterization of g-C_3_N_4_ materials [28,38]. This technique allows the determination of the combinations in which nitrogen and carbon are linked and the quantification of their ratios. In our previous work [28], a detailed discussion on the possibility of using the XPS technique in the study of g-C_3_N_4_ has been provided, and individual XPS signal assignments to specific nitrogen were proposed. There is still much controversy in the literature concerning these attributions. In general, the N 1s spectrum distinguishes four signals attributed to pyridinic nitrogen in the heptazine ring (398.6 eV; denoted Py), primary amine -NH_2_ (399.3 eV; denoted NH_2_), secondary amine -NH- (400.3 eV; denoted NH), and quaternary nitrogen originating from the N-(C)_3_ component (401.4 eV; denoted Q) [28,38,39].

**Figure 6:**
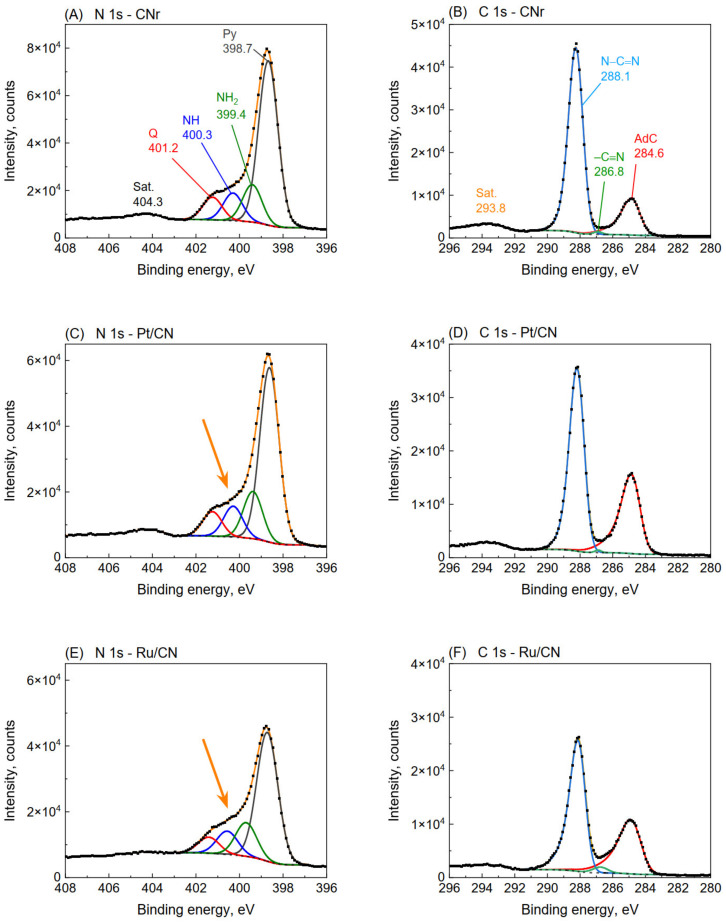
N 1s and C 1s XPS spectra of CNr (**A**,**B**), Pt/CN (**C**,**D**), and Ru/CN (**E**,**F**) catalysts (1.0 wt.%). Peaks labeled as Sat. at 293.8 and 404.3 eV are attributed to the shake-up satellites.

**Figure 7:**
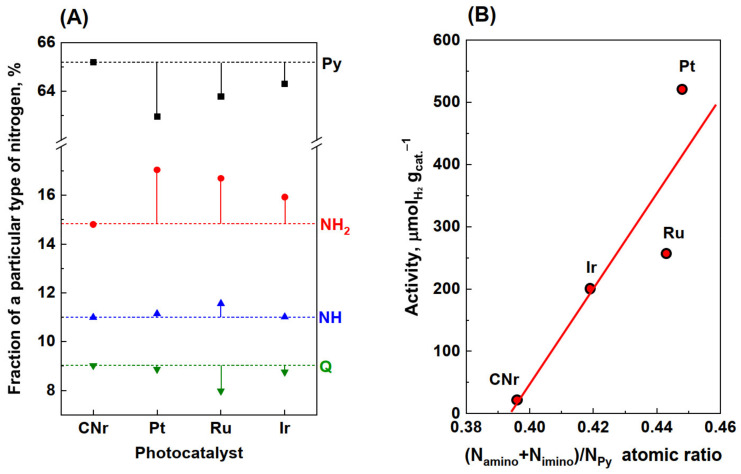
Contributions of Py, NH_2_ nitrogen, NH nitrogen, and quaternary nitrogen (Q) calculated from XPS N 1s spectra for CNr and metal photocatalysts (1.0 wt.%)—(**A**). Correlations of photocatalytic activity with the atomic ratio of sum amino- and imino-nitrogen to Py nitrogen—(**B**).

Figure 6 shows the N 1s and C 1s core-level XPS spectra for the CNr, Pt/CN, and Ru/CN samples (1.0 wt.%). The spectra of CN and CNr were practically identical; therefore, only CNr is included in Figure 6. In the N 1s spectra of CNr and metal photocatalysts, the signals originating from Py, -NH_2_, NH, and so-called quaternary (also called tertiary) nitrogen in sp^2^ hybridization could be distinguished. At first glance, the differences in the spectra of CNr and other photocatalysts seem minimal. However, closer inspection of the spectra reveals an elevation of lines in the 399–401 eV region in the Pt, Ru, and Ir catalysts compared to CNr (represented by the arrows in Figure 6C,E and Figure S3), thereby indicating an increase in the proportion of nitrogen bonded to the primary and secondary amine groups. An increase in the proportion of amine groups was observed for all metals. Based on the XPS spectra, the percentages of each nitrogen form were calculated and are shown in Figure 7A and Table S1. The most significant changes in the distribution of a variety of nitrogen were observed for the Pt/CN photocatalyst. There was a striking decrease in the proportion of Py nitrogen (over 2%), accompanied by a comparable increase in the proportion of amine nitrogen (Figure 7A). The amount of quaternary nitrogen did not change. This suggests that during the reduction of the Pt/CN photocatalyst, hydrogenolysis of N−C=N bonds in heptazine groups occurs and new −NH_2_ groups are formed at the expense of Py nitrogen. In the case of the Ir and Ru photocatalysts, an increase in the amount of −NH_2_ nitrogen was observed. It is interesting to note that for the Ru photocatalyst, the observed increase in the proportion of −NH_2_ nitrogen occurs not only at the expense of Py but also at the decrease in quaternary nitrogen. This may suggest that owing to its good hydrogenolysis properties, Ru, contradistinctively to Pt, can break the N−(C)_3_ bond. Platinum catalyzes mainly the hydrogenolysis of the aromatic N−C=N bond.

The conducted research shows that a part of the basic structural units of carbon nitride (heptazine rings) was destroyed and NH_2_ and NH groups were formed. Part of nitrogen was removed in the form of ammonia, which resulted in an increase in the C/N ratio observed in the elemental analysis. The increase in the disordered structure of carbon nitride was also confirmed by XRD analysis. The increase in disorder and a higher number of amino groups provide better charge separation, resulting in a higher photocatalytic activity [40]. This is confirmed by Figure 7B, which shows that the activity increases with respect to the amount of NH_2_ and NH nitrogen in carbon nitride. The positive effect of high temperature (550 °C) hydrogen treatment on the hydrogen evolution of carbon nitride has already been reported by Maschmeyer et al. [36]. The increase in the activity was related to the generation of structural defects associated with the delamination of g-C_3_N_4_. Similarly, it was also demonstrated that the introduction of defects in g-C_3_N_4_ nanosheets could significantly enhance the photocatalytic hydrogen evolution [36,41,42]. The large role of structural defects within g-C_3_N_4_ as water adsorption and dissociation sites was also confirmed by theoretical calculations [43].

**Figure 8:**
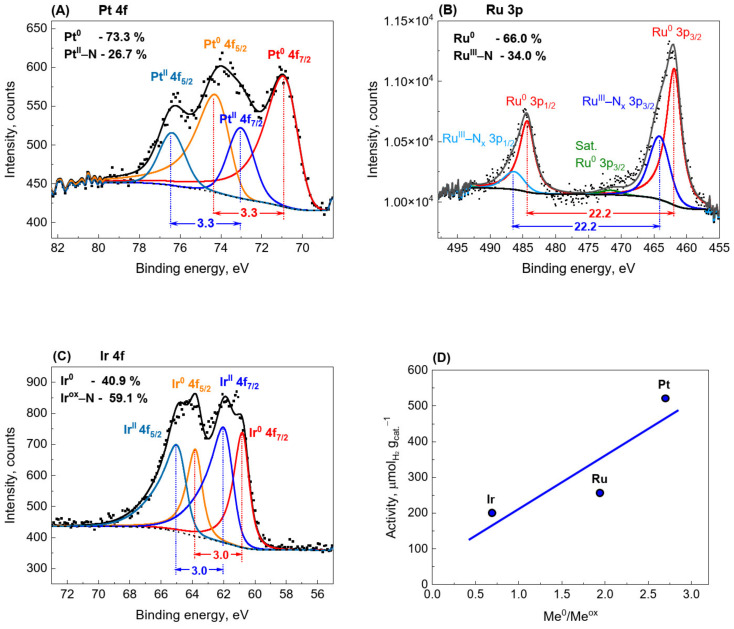
XPS detailed spectra of the reduced 1.0 wt.% photocatalysts; Pt 4f (**A**), Ru 3p (**B**), and Ir 4f (**C**). Correlations of photocatalytic activity with the ratio of metallic to an oxidized state of a metal of the reduced 1.0 wt.% photocatalysts—(**D**).

Interesting information on the catalyst structure can also be obtained from the narrow spectra of individual metals (Figure 8). The XPS spectrum of the Pt 4f region of the Pt/CN photocatalyst is shown in Figure 8A. The first doublet at 71.0 eV and 74.3 eV (with a doublet separation of 3.3 eV) corresponds to the 4f_7/2_, and 4f_5/2_ states of Pt^0^, respectively. The second doublet at 73.1 eV and 76.4 eV (with the same doublet separation of 3.3 eV) corresponds to the 4f_7/2_ and 4f_5/2_ states of platinum (II), respectively. The doublet at 73.1 eV, and 76.4 eV is often assigned to PtO. However, in this case, a signal from lattice oxygen should be visible in the O 1s spectrum, which generally occurs at 530.5–530.8 eV [44,45,46,47]. Meanwhile, such a signal in the O 1s spectrum was not observed (Figure S4); hence, the presence of PtO can be excluded. However, similar bands are also characteristic of the platinum (II) complex with phthalocyanine [48], for which a Pt 4f_7/2_ signal was observed at 73.2 eV [48]. Similarly, in a study by Battistoni et al. [49] on platinum (II) complexes of methyl esters of dithiocarbazic acid, bands at 73.3 ± 0.2 and 76.6 ± 0.2 eV corresponding to Pt 4f_7/2_ and Pt 4f_5/2_, respectively, were reported. The average separation of the doublets was 3.31 eV. In the present work, the positions of the bands corresponding to Pt 4f_7/2_ and Pt 4f_5/2_ are at 73.1 and 76.4 eV, respectively, with the separation of 3.3 eV. The almost perfect correspondence between the positions of the signals and the magnitude of their separation suggests that we are dealing with Pt in a +2 oxidation state coordinated to nitrogen atoms from g-C_3_N_4_. Such combinations were recently observed by Zhang et al. [50,51] for single-atom Pt/C_3_N_4_ photocatalysts. In addition, theoretical calculations confirmed the possibility of Pt−N bond formation [51,52,53]. Quantitative analysis of the XPS spectra showed that the proportion of Pt coordinated with nitrogen atoms (Pt^ox^−N_x_) was 26.7%, and the rest was metallic Pt (73.3%). The analysis of the Ir 4f spectra led to similar conclusions (Figure 8C). The Ir 4f spectrum presents two chemical states of Ir: the emission peaks at 60.8 and 63.8 eV correspond to the typical values of 4f_7/2_ and 4f_5/2_ photoelectrons of metallic Ir^0^, respectively [54]. The second doublet at 62.2 and 65.2 eV is generally attributed to the iridium oxides [55,56]. However, similar to the Pt photocatalysts discussed above, we did not observe signals characteristic of iridium oxides in the O 1s spectrum (Figure S4), which occur at 530.0 eV and 530.5 eV for anhydrous and hydrated IrO_2_, respectively [55]. Vasapollo et al. [57] reported an Ir 4f_7/2_ signal for nitrosoarene complex iridium (III) at 62.1 eV. Furthermore, Hu and Chu [58] obtained an Ir 4f_7/2_ signal for Ir-doped polyaniline films at 61.6 eV, which is attributed to Ir–N coupling. In view of the above, we are inclined to interpret that the 62.2 and 64.0 eV doublet originates from Ir at higher oxidation states (+3 and/or +4) bonded to nitrogen atoms. Iridium coordinated to nitrogen atoms (Ir^ox^−N_x_) accounted for 59.1%, and the remaining 40.9% consisted of metallic Ir.

Figure 8B shows Ru 3p core-level spectra of the Ru/CN photocatalyst. The doublet at 461.9 and 484.1 eV with a doublet separation of 22.2 eV originates from metallic Ru [59]. The second doublet (at 464.1 and 483.3 eV) with the same splitting as that for Ru^0^, has several characteristics of the nitrosyl, aryldiazo, and aryldiimine complexes of Ru [60] or [Ru^II^(NH_3_)_5_(N_2_)]Cl_2_ (pentaammine(dinitrogen)ruthenium dichloride) [61]. Hence, it can be assumed with a high probability that these signals originate from Ru coordinated to nitrogen atoms in the carbon nitride [62]. As in the case of Pt and Ir, the presence of ruthenium oxides can be excluded because the characteristic O 1s signals originating from lattice oxygen (O 1s for RuO_2_ BE = 529.3 eV) were not observed (Figure S4) [59]. The proportions of metallic Ru and Ru^ox^−N_x_ were 66.0% and 34.0%, respectively.

The XPS results show that upon reduction of the photocatalyst, metallic nanoparticles are formed that are closely bonded to the carbon nitride surface by a transient layer of oxidized metal coordinated to nitrogen atoms and/or amine groups. The formation of such an intermediate layer was recently observed by Benisti et al. [40] for Pt photocatalysts deposited on g-C_3_N_4_ hollow nanospheres. The metallic nanoparticles constitute an excellent reservoir of photodegraded electrons, and the intermediate oxidized layer facilitates their transport from the carbon nitride surface [40]. This has a beneficial effect on hydrogen evolution, as confirmed by the relationship shown in Figure 8D, illustrating the effect of the Me^0^/Me^ox^ ratio on photocatalytic activity.

## 4. Conclusions

In summary, it has been shown that in a reducing atmosphere at 300 °C, selective etching of the carbon nitride structure occurs through hydrogenolysis and hydrogenation of C−N bonds by metal nanoparticles. A part of the heptazine rings (basic structural units of carbon nitride) was destroyed and new NH_2_ and NH groups were formed. Etching of g-C_3_N_4_ leads to partial elimination of nitrogen and to an increase in specific surface area. Metallic nanoparticles are closely bonded to the carbon nitride surface by a transient layer of oxidized metal coordinated to nitrogen atoms. All the above changes come down to an increase in the deformation of the g-C_3_N_4_ structure, which favors the better separation of charges and, as a result, higher activity in the production of hydrogen through the water-splitting reaction.

## Figures and Tables

**Table 1:** Sizes of the g-C_3_N_4_ crystallites calculated using the Scherrer formula based on the (002) reflex for CN, CNr, and 1.0 wt.% metal photocatalysts.

Sample	Peak Position 2θ (deg)	FWHM (deg)	Interplanar Distance (nm)	Crystallite Size (nm)	Number of Layers
CN	27.616	0.8383	0.323	10.20	31.6
CNr	27.602	0.8331	0.323	10.26	31.7
Pt/CN	27.586	0.9024	0.323	9.47	29.3
Ru/CN	27.806	0.9182	0.321	9.32	29.1
Ir/CN	27.669	0.9188	0.322	9.31	28.9

**Table 2:** Elemental analysis of CNr and 1.0 wt.% metal photocatalysts.

Sample	At.%				C/N
	N	C	H	O	
CNr	45.94	32.13	19.04	2.89	0.70
Pt/CN	45.44	33.81	18.52	2.23	0.74
Ru/CN	41.70	31.51	24.98	1.81	0.76
Ir/CN	45.10	33.39	19.67	1.84	0.74

## Data Availability

The data presented in this study are available on request from the corresponding author.

## Supplementary Materials

The following are available online at https://www.mdpi.com/article/10.3390/ma15030710/s1, Figure S1. The specific surface area of carbon nitride and photocatalysts with 0.5 wt.% metal loading—(A). Cumulative pore volume and average pore size for 0.5 wt.% photocatalysts—(B). Figure S2. SEM images of supports and photocatalysts; Figure S3. XPS spectra of N 1s region for reduced carbon nitride and photocatalyst samples. The spectra were normalized relative to the most intense contribution to expose spectral variations in the 397–403 eV range; Figure S4. XPS core level spectra of the O 1s for gCNr and metal/CN photocatalysts. The O1s signal at 532.2eV is from adsorbed oxygen. The signal at ~533.5 eV comes from adsorbed water; Table S1. XPS results for CN, CNr, and metal photocatalysts (1.0 wt.% of metals).
