# Supplementary material for: Reductive Modification of Carbon Nitride Structure by Metals—The Influence on Structure and Photocatalytic Hydrogen Evolution
Source: Materials (Basel). 2022 Jan 18;15(3):710. doi: 10.3390/ma15030710 (PMC8836795; doi:10.3390/ma15030710)
Supplement: Supplementary file 1 [file materials-15-00710-s001.zip › materials-1556495-supplementary.pdf]

Type of the Paper (Article, Review, Communication, etc.)

Reductive modification of carbon nitride structure by metals - the influence on structure and photocatalytic hydrogen evolution

Emilia Alwin [1], Robert Wojcieszak [2], Kamila Kočí [3], Miroslava Edelmannová [3], Michał Zieliński [1], Agata Suchora [1], Tomasz Pędziński [1,4] and Mariusz Pietrowski*[1]

[1] Faculty of Chemistry, Adam Mickiewicz University, Poznań, Uniwersytetu Poznańskiego 8, 61-614 Poznan, Poland; emilia.alwin@amu.edu.pl (E.A.); agasuc@amu.edu.pl (A.S.); mardok@amu.edu.pl (M.Z.); tomekp@amu.edu.pl (T.P.)

[2] Univ. Lille, CNRS, Centrale Lille, Univ. Artois, UMR 8181 - UCCS - Unité de Catalyse et Chimie du Solide, F-59000 Lille, France; robert.wojcieszak@univ-lille.fr (R.W.)

[3] Institute of Environmental Technology, CEET, VSB-Technical University of Ostrava, 17. listopadu 15/2172, Ostrava-Poruba, 70800, Czech Republic; kamila.koci@vsb.cz (K.K.); miroslava.edelmannova@vsb.cz (M.E.)

[4] Centre for Advanced Technologies, Adam Mickiewicz University, Poznań, Uniwersytetu Poznańskiego 10, 61-614 Poznan, Poland; tomekp@amu.edu.pl (T.P.)

* Correspondence: mariop@amu.edu.pl (M.P.)

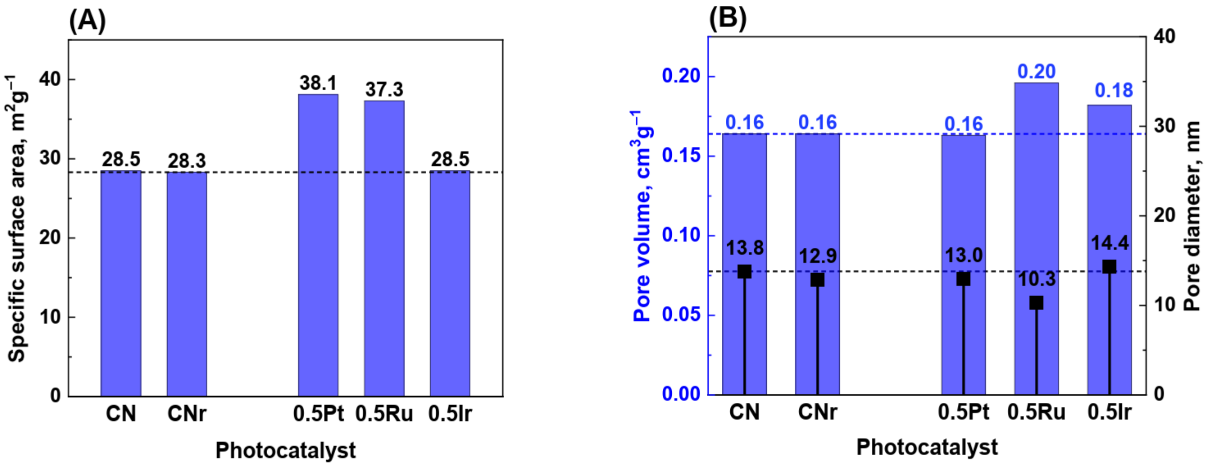

Figure S1. Specific surface area of carbon nitride and photocatalysts with 0.5 wt.% metal loading - (A). Cumulative pore volume and average pore size for 0.5 wt.% photocatalysts - (B).

CN

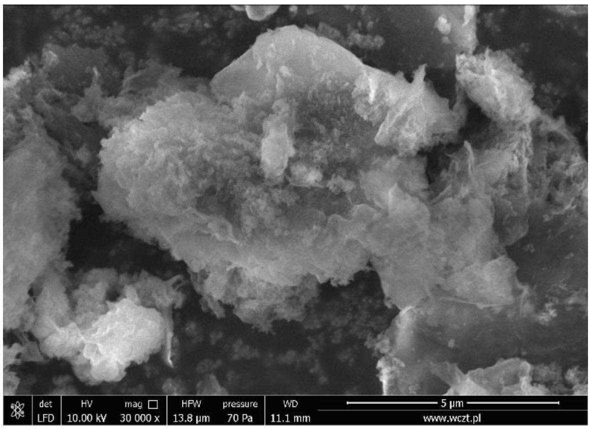

CNr

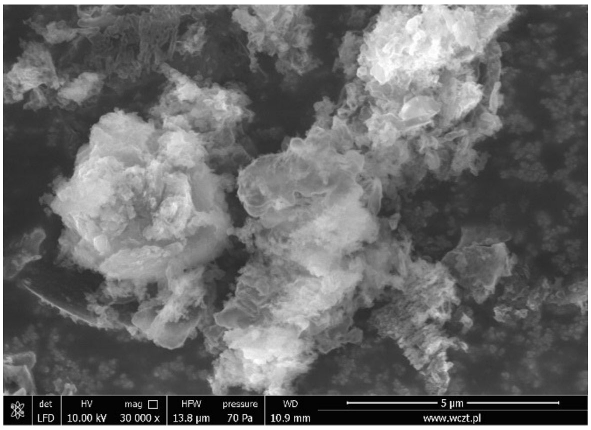

Pt/CN

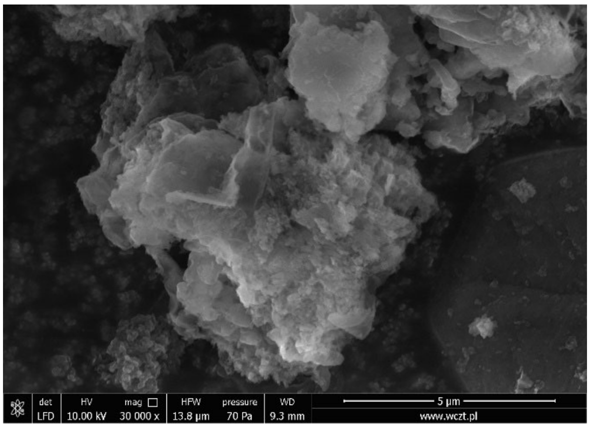

Ru/CN

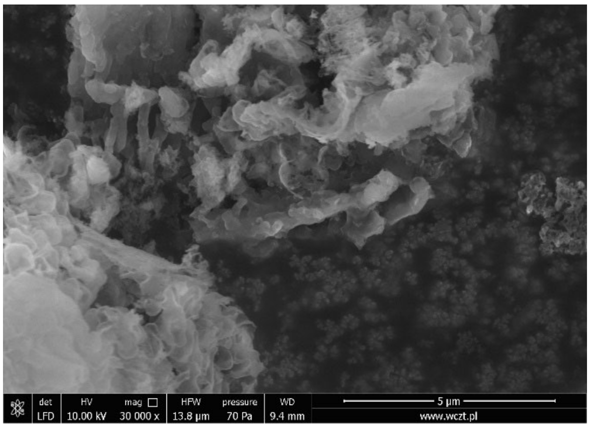

Ir/CN

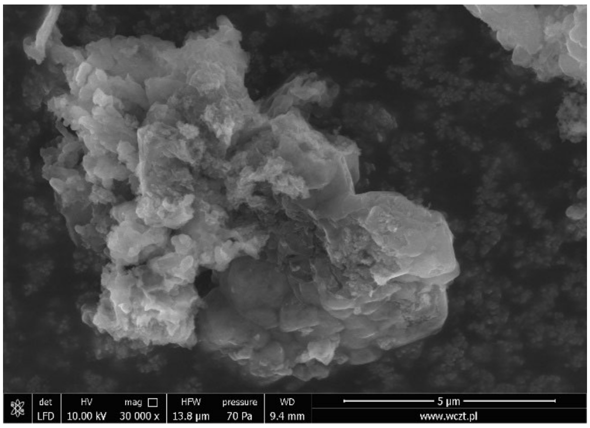

Figure S2. SEM images of supports and 1.0 wt.% photocatalysts.

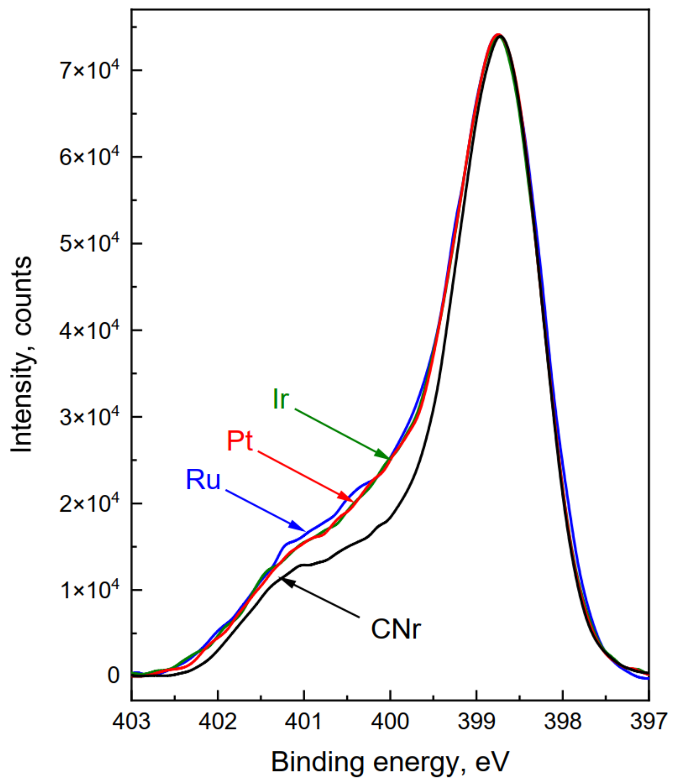

Figure S3. XPS spectra of N 1s region for reduced carbon nitride and photocatalyst samples (1.0 wt.%). The spectra were normalized relatively to the most intense contribution to expose spectral variations in the 397-403 eV range.

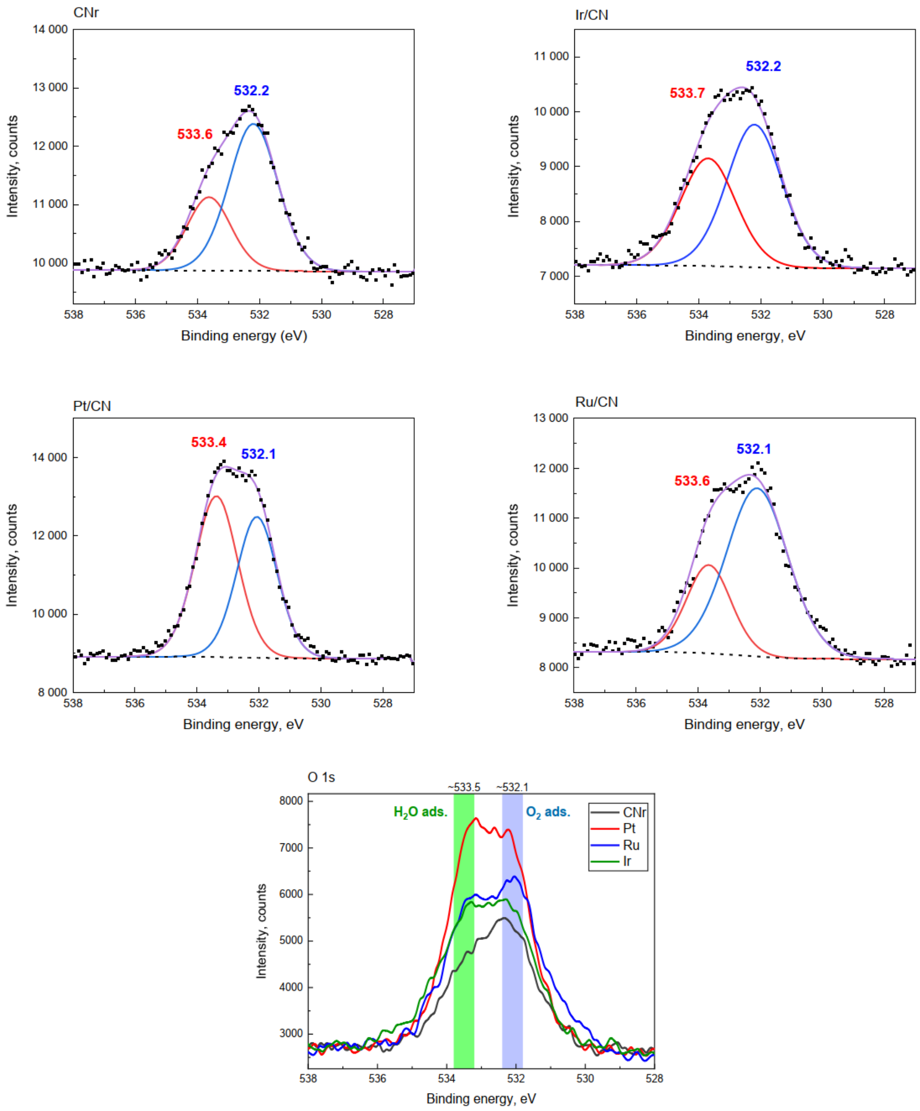

Figure S4. XPS core level spectra of the O 1s for gCNr and metal/CN photocatalysts. The O1s signal at 532.2eV is from adsorbed oxygen [1]. The signal at ~533.5 eV come from adsorbed water [2].

Table S1. XPS results for CN, CNr, and metal photocatalysts (1.0 wt.% of metals).

	CNr				Pt/CN				Ru/CN				Ir/CN			
	**B.E., eV**	**FWHM**	**%at.**	**%N**	**B.E., eV**	**FWHM**	**%at.**	**%N**	**B.E., eV**	**FWHM**	**%at.**	**%N**	**B.E., eV**	**FWHM**	**%at.**	**%N**
**Py**	398.68	1.03	31.36	65.2	398.62	1.02	26.04	62.96	398.72	1.13	25.7	63.78	398.57	1.1	25.93	64.3
**NH2**	399.30	1.03	7.14	14.8	399.38	1.02	7.05	17.04	399.7	1.13	6.73	16.69	399.5	1.1	6.42	15.9
**NH**	400.3	1.03	5.29	11.0	400.29	1.02	4.61	11.14	400.56	1.13	4.66	11.56	400.28	1.1	4.44	11.0
**Quaternary**	401.24	1.03	4.34	9.0	401.25	1.02	3.66	8.86	401.41	1.13	3.21	7.97	401.17	1.1	3.53	8.8
**Total nitrogen:**			**48.13**				**41.36**				**40.3**				**40.32**	
**C-C**	284.59	1.23	10.89		284.6	1.24	20.81		284.6	1.3	21.53		284.6	1.46	20.8	
**C≡N**	286.83	0.59	0.34		286.84	0.38	0.18		286.85	1.31	1.2		286.68	1.04	1.34	
**N=C-N (Ar)**	288.14	1.02	38.88		288.04	1.02	34.32		288.05	1.07	33.24		287.93	1.04	34.2	
**Carbon in C-N:**			**39.22**				**34.5**				**34.44**				**35.54**	
**Oxygen 1**	532.59	2.52	1.75		532.07	1.48	1.54		532.22	2.56	3.11		531.84	1.23	0.42	
**Oxygen 2**					533.36	1.54	1.79		533.73	1.39	0.61		533.06	2.72	2.92	

References

1. Yang L.; Zhou X.; Song L.; Wang Y.; Wu X.; Han N.; Chen Y. Noble Metal/Tin Dioxide Hierarchical Hollow Spheres for Low-Concentration Breath Methane Sensing. *ACS Appl. Nano Mater.* **2018**, *1*, 6327-6336.
2. Peuckert M. XPS investigation of surface oxidation layers on a platinum electrode in alkaline solution. *Electrochim. Acta* **1984**, *29*, 1315-1320.
